# Thin endometrium is associated with the risk of hypertensive disorders of pregnancy in fresh IVF/ICSI embryo transfer cycles: a retrospective cohort study of 9,266 singleton births

**DOI:** 10.1186/s12958-021-00738-9

**Published:** 2021-04-09

**Authors:** Xiaojie Liu, Jingwan Wang, Xiao Fu, Jing Li, Meng Zhang, Junhao Yan, Shanshan Gao, Jinlong Ma

**Affiliations:** 1 Cheeloo College of Medicine, Shandong University, Jinan, China; 2 Center for Reproductive Medicine, Shandong University, No.157 Jingliu Road, Jinan, 250021 China; 3 National Research Centre for Assisted Reproductive Technology and Reproductive Genetics, Shandong University, Jinan, China

**Keywords:** Endometrial thickness, Hypertensive disorders of pregnancy, Fresh IVF/ICSI embryo transfer, Obstetric complication, Perinatal outcome

## Abstract

**Background:**

Thin endometrial thickness (EMT) has been suggested to be associated with reduced incidence of pregnancy rate after in vitro fertilization/intracytoplasmic sperm injection (IVF/ICSI) treatment, but the effect of thin endometrium on obstetric outcome is less investigated. This study aims to determine whether EMT affects the incidence of obstetric complications in fresh IVF/ICSI-embryo transfer (ET) cycles.

**Methods:**

We conducted a retrospective cohort study collecting a total of 9266 women who had singleton livebirths after fresh IVF/ICSI-ET treatment cycles at the Center for Reproductive Medicine Affiliated to Shandong University between January 2014 and December 2018. The women were divided into three groups according to the EMT: 544 women with an EMT ≤8 mm, 6234 with an EMT > 8–12 mm, and 2488 with an EMT > 12 mm. The primary outcomes were the incidence of obstetric complications including hypertensive disorders of pregnancy (HDP), gestational diabetes mellitus (GDM), placental abruption, placenta previa, postpartum hemorrhage (PPH) and cesarean section. Multivariable logistic regression analysis was performed to calculate the odds ratios (ORs) and 95% confidence intervals (CIs) for associations between the EMT measured on the day of human chorionic gonadotropin (HCG) trigger and the risk of the outcomes of interest.

**Results:**

The HDP incidence rate of pregnant women was highest in EMT ≤ 8 mm group and significantly higher than those in EMT from > 8–12 mm and EMT > 12 mm group, respectively (6.8% versus 3.6 and 3.5%, respectively; *P* = 0.001). After adjustment for confounding variables by multivariate logistic regression analysis, a thin EMT was still statistically significant associated with an increased risk of HDP. Compared with women with an EMT > 8–12 mm, women with an EMT ≤8 mm had an increased risk of HDP (aOR = 1.853, 95% CI 1.281–2.679, *P* = 0.001).

**Conclusion:**

A thin endometrium (≤8 mm) was found to be associated with an increased risk of HDP after adjustment for confounding variables, indicating that the thin endometrium itself is a risk factor for HDP. Obstetricians should remain aware of the possibility of HDP when women with a thin EMT achieve pregnancy through fresh IVF/ICSI–ET treatment cycles.

**Supplementary Information:**

The online version contains supplementary material available at 10.1186/s12958-021-00738-9.

## Background

Assisted reproductive technology (ART) has been widely applied in infertility treatment and the technology advance has greatly increased the live birth rate in recent years [1, 2]. Therefore, achieving healthy perinatal outcome became a new focus reproductive medicine. Numerous studies have shown that compared with spontaneous conception, ART pregnancies are associated with increased risks for obstetric complications such as preeclampsia, gestational diabetes mellitus (GDM) and placental anomalies [3–6]. It is still unclear what risk factors in ART cycles may be related to these adverse obstetric complications. A study by Wang YA et al. and a later meta-analysis revealed that this may be attributed to a higher frequency of multiple gestations after ART [7, 8]. But another systematic review and meta-analysis has shown that women with singleton ART pregnancies still have a higher incidence of obstetric and perinatal complications compared with their counterparts with spontaneous conception [9].

The embryo quality and endometrial receptivity play an important role in fresh in vitro fertilization/intracytoplasmic sperm injection-embryo transfer (IVF/ICSI–ET) pregnancy outcome. Endometrial receptivity is the ability of an endometrium allowing normal implantation [10]. Meanwhile, the endometrial thickness (EMT) is considered as one of the key factors influencing endometrial receptivity [11]. Several cohort studies demonstrated that the EMT on the day of human chorionic gonadotropin (HCG) administration was related to IVF/ICSI pregnancy rates and was regarded as a possible predictor for pregnancy outcomes [12–14]. A recent systematic review and meta-analysis showed that the implantation rates, pregnancy rates, ongoing pregnancy rates, and live birth rates in women with a lower EMT were reduced compared with those with a higher EMT [15]. In addition, an EMT of less than 7.5 mm was found to be associated with increased risks of adverse obstetric outcomes in a retrospective cohort study of 864 singleton deliveries [16].

Only a few studies have explored the association between EMT and obstetric outcomes after IVF/ICSI–ET, with controversial results, and the sample sizes of most previous research were limited (generally less than 5000). Therefore, we performed this retrospective cohort study including 9266 fresh embryo transfer (ET) cycles with singleton birth after controlling some pregnancy complication-related factors. This study aimed to provide an up-to-date evidence to determine a more accurate relationship between EMT measured on the day of HCG trigger in fresh IVF/ICSI–ET cycles and the risk of obstetric complications including hypertensive disorders of pregnancy (HDP), GDM, placental abruption, placenta previa, postpartum hemorrhage (PPH) and cesarean section.

## Methods

### Study design and population

This is a retrospective cohort study conducted at the Center for Reproductive Medicine Affiliated to Shandong University. The study protocol was approved by the Institutional Review Board of Center for Reproductive Medicine Affiliated to Shandong University. We extracted data of all live births following fresh IVF/ICSI-ET treatment cycles in our reproductive center between January 2014 and December 2018. Our inclusion criteria were: [1] patients with a singleton birth, [2] patients under the age of 45 years. Exclusion criteria were: [1] patients undergoing preimplantation genetic testing (PGT) cycles, oocyte donation cycles, or frozen embryo transfer (FET) cycles, [2] patients with chronic hypertension or diabetes mellitus before the index pregnancy, [3] patients with multiple births, [4] patients with congenital or acquired uterine malformations. [5] patients who were diagnosed with intrauterine disease (e.g., endometrial polyps, uterine fibroids, intrauterine adhesion), and regardless of whether they have received surgical treatment or not. Finally, a total of 9266 women under the age of 45 years were included in this study.

### IVF/ICSI protocols

The methods of ovarian stimulation protocols and ET protocols in our reproduction medicine center have been described in detail elsewhere [17, 18]. There were several stimulation protocols in our study: gonadotropin-releasing hormone (GnRH) agonist long protocols (4904 cycles, 52.92%), GnRH agonist short protocols (2211 cycles, 23.86%), flexible GnRH antagonist protocols (1660 cycles, 17.91%), GnRH agonist super long protocols (424 cycles, 4.58%), and other protocols (67 cycles, 0.72%). Other protocols are improvement protocols based on the individual treatment. The selection of different ovarian stimulation protocols was depending on patients’ individual conditions, including patient’s age, body weight, infertility causes, and ovarian function. HCG at a dose of 4000 to 10,000 IU was administrated intramuscularly to induce oocyte maturation when two or more follicles reached a mean diameter of 18 mm. The dual trigger was also applied in portion of GnRH antagonist protocols. Transvaginal ultrasound-guided oocyte retrieval was carried out 34 to 38 h after HCG administration. For patients who received fresh ET in our center, oral dydrogesterone and utrogestan vaginal capsules were used for lutealphase support, which was started immediately on the day of oocyte retrieval and was continued until 10 weeks of conception. Fertilization of the oocytes were performed by IVF or ICSI according to the male partner’s sperm quality. Usually, clinicians in our reproductive center recommend ICSI instead of conventional IVF or intrauterine insemination (IUI) if sperm concentration below 5*10^6^/ml, or sperm morphology (normal forms) results of below 5%, or sperm progressive motility below 10% (a + b grade). High-quality embryos were picked out for cleavage-stage embryo or blastocyst transfer at 3 or 5 days after fertilization, respectively.

### Endometrial thickness assessment

The thickness of endometrium was measured to the nearest 0.1 mm by doctors highly trained in ultrasound monitoring of the same team. These doctors used the same ultrasound machines with intracavity probe according to the same standardized protocols in our department (Toshiba Color Ultrasound Aplio 500, Toshiba Co., Ltd., Japan). Thickness was measured in the midsagittal plane as the maximal distance from one interface of endometrial-myometrial to the other wall of the uterus. EMT on the day of ovulation trigger was recorded and used for statistical analysis in this research. Patients were divided into three groups depending on EMT: ≤8 mm, > 8–12 mm, and ≥ 12 mm, which was consistent with previous studies [17, 19, 20]. In addition, the optimal cut-off threshold for a thin endometrium to discriminate HDP is 8.08 mm with an Area Under the Curve of 61.2% in a receiver operating characteristic curve (Fig. S1).

### Patients’ follow up

In the early pregnancy, patients return to our reproductive centre for serum or ultrasound examinations. The 1st follow-up was performed around 14th days after embryo transfer, and biochemical pregnancy was assessed by measuring the serum level of HCG-beta subunit. The 2nd follow-up was performed at 5 and 6 weeks after embryo transfer, and clinical pregnancy was detected with the confirmation of gestational sacs by transvaginal. Ongoing pregnancy was confirmed at the 3rd follow-up, which was performed at 12th week of gestation (around 9 to 10 weeks after ET). Subsequent prenatal care was then performed in the obstetric department. The patients received telephone surveys by trained nurses in perinatal period, and standardized questionnaires were used to collect information including perinatal complications, gestational weeks, birth date, delivery mode, newborn gender and birth weight, neonatal diseases, treatment and prognosis. To avoid potential bias, the patients would be defined as loss of follow-up after at least five times failed attempts to contact by telephone. The follow-up information was recorded in detail and then saved together with previous treatment information in the electronic medical records. The data of this research were extracted from the electronic database of our hospital and a total of 137 patients (1.5% of all subjects who met the inclusion criteria) lost to follow-up or with core data missing in the electronic data base were excluded.

### Outcome measures

The primary outcomes of our study were HDP, GDM, placental abruption, placenta previa, postpartum hemorrhage (PPH) and cesarean section. Live birth was defined as the delivery of a live-born infant after 28 weeks gestation. HDP included gestational hypertension (blood pressure ≥ 140/90 mmHg at least two occasions more than 4 h apart after 20 weeks’ gestation), preeclampsia (gestational hypertension and the coexistence of one or both of the following new-onset conditions: proteinuria; other maternal organ dysfunction), and eclampsia [21]. In this research, we excluded patients with chronic hypertension—which may be diagnosed before pregnancy or in the early stages of pregnancy (< 20 weeks’ gestation) [21]. GDM was defined as any degrees of abnormal glucose metabolism during pregnancy that was not clearly overt diabetes prior to gestation [22]. The 75-g OGTT (oral glucose tolerance test) was performed for woman at 24–28 weeks of gestation, with plasma glucose measurement when she is fasting (in the morning after an overnight fast of at least 8 h) and at 1 and 2 h. The diagnosis of GDM is made when any of the following plasma glucose values are met or exceeded: fasting: 92 mg/dL (5.1 mmol/L); 1 h: 180 mg/dL (10.0 mmol/L); 2 h: 153 mg/dL (8.5 mmol/L) [22]. Placenta previa was defined as an abnormal location of the placenta over or in close proximity to the internal cervical os [23]. Placental abruption was defined as a premature separation of the placenta before delivery [24]. According to the American College of Obstetrician and Gynecologists (ACOG) practice bulletin, postpartum hemorrhage was defined as cumulative blood loss ≥1000 mL or blood loss accompanied by symptoms of hypovolemia within 24 h after childbirth regardless of delivery mode [25].

### Statistical analysis

Continuous data were represented as mean ± standard deviation, and were compared by one-way analysis of variance (ANOVA) or Welch’s ANOVA. Welch’s ANOVA was used if the data failed the assumption of equal variances. Otherwise, the one-way ANOVA was used. Categorical data are represented as frequencies and percentages, and univariate analysis including Pearson chi-square test or Fisher’s exact test was used to compare the distribution among groups. A logistic regression model incorporating restricted cubic splines (RCSs) was performed for the multivariate analysis to calculate the odds ratios (ORs) and 95% confidence intervals (CIs) for associations between the EMT measured on the day of HCG trigger and the risk of HDP. Adjusted variables included age, body mass index (BMI), estradiol and progesterone level on HCG administration day, parity, type of ART treatment, protocol for controlled ovarian stimulation, type of infertility, number of embryos transferred, stage of embryo transferred, previous cesarean section, endometriosis, and polycystic ovary syndrome (PCOS). Endometriosis was defined as the presence of endometrial tissue outside the uterine cavity [26]. The definition of PCOS was based on the Rotterdam definition—that PCOS can be diagnosed in woman existing of at least two of the three following characteristics: clinical and/or biochemical hyperandrogenism, ovulatory dysfunction and polycystic ovarian morphology [27, 28]. For variables of age, BMI, estradiol and progesterone level on HCG administration day, RCSs were generated to adjust for the non-linear relationship between these continuous variables and the outcomes of interest. When modelling RCS, knots location of the spline was tested according to Harrell’s recommendations [29], and model fitting was assessed on the basis of the Akaike information criterion (AIC). Then, models with knots placed at the 5th, 35th, 65th, and 95th percentile of these four continuous variables were proved to be optimal based on the minimum value of AIC. A *P*-value of less than 0.05 was considered statistically significant. All statistical analyses were performed with the Statistical Package for the Social Sciences (version 24.0; SPSS Inc., USA) and Statistics Analysis System (version 9.4; SAS Institute Inc., USA).

## Results

During our study period, 9266 singletons born following fresh IVF/ICSI-ET cycles met our study criteria, including 544 women with an EMT ≤8 mm, 6234 with an EMT > 8–12 mm, and 2488 with an EMT > 12 mm, and we defined the women with an EMT > 8–12 mm as a reference group. The demographic and main treatment characteristics are presented in Table 1. Mean endometrial thicknesses were 7.57 ± 0.71 mm, 10.52 ± 1.06 mm, and 13.38 ± 1.11 mm in the EMT ≤8, > 8–12 and > 12 mm groups, respectively. Statistically significant differences were found in age, parity, the type and cause of infertility, ovarian stimulation protocol, progesterone level on HCG trigger day and the number of embryos transferred among the three groups, as shown in Table 1.

**Table 1:** Demographic and main treatment characteristics for women with singleton live births according to endometrial thickness

Characteristics	EMT ≤ 8 mm	8 < EMT ≤ 12 mm	EMT > 12 mm	***P*** value
	(*n* = 544)	(*n* = 6234)	(*n* = 2488)	
Age (y)^abc^	32.39 ± 4.43	30.91 ± 4.30	30.46 ± 4.33	< 0.001^e^
BMI (kg/m^2^)	23.55 ± 3.36	23.50 ± 3.55	23.69 ± 3.65	NS^e^
Parity, n (%) ^ab^				< 0.001^f^
First	362 (66.5)	4800 (77.0)	1912 (76.8)	
High order	182 (33.5)	1434 (23.0)	576 (23.2)	
Primary infertility, n (%) ^abc^	167 (30.7)	2982 (47.8)	1340 (53.9)	< 0.001^f^
Cause of infertility, n (%) ^abc^				< 0.001^f^
Oviduct factors	241 (44.3)	1844 (29.6)	668 (26.8)	
Male factor	128 (23.5)	1749 (28.1)	745 (29.9)	
Ovulation problems	20 (3.7)	321 (5.1)	120 (4.8)	
Multiple causes	43 (7.9)	896 (14.4)	364 (14.6)	
Unexplained	67 (12.3)	872 (14.0)	351 (14.1)	
Other	45 (8.3)	552 (8.9)	240 (9.6)	
Type of ART treatment, n (%)				NS^f^
IVF	396 (72.8)	4476 (71.9)	1755 (70.5)	
ICSI	148 (27.2)	1758 (28.2)	733 (29.5)	
Ovarian stimulation protocol, n (%) ^c^				< 0.001^f^
GnRH antagonist protocol	102 (18.8)	1153 (18.5)	405 (16.3)	
GnRH agonist long protocol	202 (37.1)	3183 (51.1)	1519 (61.1)	
GnRH agonist short protocol	187 (34.4)	1580 (25.3)	444 (17.8)	
GnRH agonist super long protocol	37 (6.8)	275 (4.4)	112 (4.5)	
Other protocol	16 (2.9)	43 (0.7)	8 (0.3)	
EMT on HCG trigger day, (mm)^abc^	7.57 ± 0.71	10.52 ± 1.06	13.38 ± 1.11	< 0.001^d^
Progesterone level on HCG trigger day (ng/mL) ^ac^	0.77 ± 0.37	0.82 ± 0.35	0.80 ± 0.34	0.001^e^
E_2_ level on HCG trigger day (pg/mL)	2856.69 ± 1442.43	2878.68 ± 1431.83	2952.69 ± 1455.60	NS^e^
Number of embryos transferred, n (%) ^ab^				0.002^f^
1	210 (38.6)	1952 (31.3)	782 (31.4)	
2	334 (61.4)	4282 (68.7)	1706 (68.6)	
Blastocyst transfer, n (%)	165 (30.3)	1603 (25.7)	632 (25.4)	0.05^f^

Values are presented as mean ± standard deviation or n (%). *ANOVA* analysis of variance, *BMI* body mass index, *EMT* endometrial thickness, *HCG* human chorionic gonadotropin, *E*_*2*_ estradiol, *NS* not statistically significant

^a^Statistically significant differences between EMT ≤8 and 8 < EMT ≤12 mm groups

^b^Statistically significant differences between EMT ≤8 mm and EMT > 12 mm groups

^c^Statistically significant differences between 8 < EMT ≤12 mm and EMT > 12 mm groups

^d^Welch’s ANOVA

^e^One-way ANOVA

^f^Pearson chi-square test

Obstetric complications for women with singleton live births according to EMT groups are presented in Table 2. The HDP incidence rate was highest in women with an EMT ≤8 mm and significantly higher than those in women with an EMT > 8–12 mm and EMT > 12 mm, respectively (6.8% versus 3.6 and 3.5%, respectively; *P* = 0.001). In addition, there were no statistically significant differences in rates of GDM, placental abruption, placenta previa, PPH and cesarean section among the three groups. We further performed univariate regression analysis of variables which were potential predictors of the incidence of HDP, as shown in Table 3. The risk of HDP was statistically significant increased in the EMT ≤ 8 mm group compared with those from the EMT > 8–12 mm group (OR 1.958; 95% CI, 1.367–2.805; *P* < 0.001), and there was no statistically significant difference in the incidence of HDP between the EMT > 8–12 and > 12 mm groups. At the same time, maternal age, BMI, and previous cesarean section were also potential predictors for HDP. A multivariate logistic regression incorporating RCSs was subsequently conducted and EMT was still significantly associated with HDP after adjusting for confounding variables (Table 3). Compared with women with an EMT > 8–12 mm, women with an EMT ≤8 mm had an adjusted odds ratio (aOR) of 1.853 (95% CI, 1.281–2.679, *P* = 0.001), and we found no statistically significant difference between the EMT > 8–12 and > 12 mm groups.

**Table 2:** Obstetric complications for women with singleton live births according to endometrial thickness

Outcomes	EMT ≤ 8 mm	8 < EMT ≤ 12 mm	EMT > 12 mm	***P*** value
	(*n =* 544)	(*n =* 6234)	(*n =* 2488)	
HDP, n (%) ^ab^	37 (6.8)	224 (3.6)	88 (3.5)	0.001^c^
GDM, n (%)	52 (9.6)	469 (7.5)	187 (7.5)	0.224 ^c^
Placental abruption, n (%)	4 (0.7)	15 (0.2)	11 (0.4)	0.054 ^d^
Placenta previa, n (%)	10 (1.8)	81 (1.3)	43 (1.7)	0.232 ^c^
PPH, n (%)	2 (0.4)	19 (0.3)	7 (0.3)	0.894 ^d^
Cesarean section, n (%)	346 (63.6)	3862 (62.0)	1526 (61.3)	0.604 ^d^

*HDP* hypertensive disorders of pregnancy; HDP include gestational hypertension, preeclampsia, and eclampsia, *GDM* gestational diabetes mellitus, *PPH* postpartum hemorrhage

^a^Statistically significant differences between EMT ≤8 mm and 8 mm < EMT ≤12 mm groups

^b^Statistically significant differences between EMT ≤8 mm and EMT > 12 mm groups

^c^Pearson chi-square test

^d^Fisher’s exact test

**Table 3:** Univariate and multivariate analysis of predictor variables for HDP

Predictor variable	OR (95% CI)	*P* value	aOR (95% CI)	*P* value
EMT on HCG trigger day (mm)				
≤ 8	1.958 (1.367–2.805)	< 0.001^a^	1.853 (1.281–2.679)	0.001^a^
> 8–12	1		1	
> 12	0.984 (0.765–1.265)	0.898	1.000 (0.775–1.291)	0.999
Parity				
First	1		1	
High order	1.007 (0.783–1.295)	0.955	0.774 (0.550–1.088)	0.141
ART treatment typy				
IVF	1		1	
ICSI	0.923 (0.725–1.174)	0.514	0.961 (0.751–1.229)	0.751
Ovarian stimulation protocol				
GnRH antagonist protocol	1		1	
GnRH agonist long protocol	1.004 (0.740–1.362)	0.981	1.071 (0.784–1.463)	0.667
GnRH agonist short protocol	1.346 (0.967–1.875)	0.078	1.205 (0.848–1.712)	0.298
GnRH agonist super long protocol	1.319 (0.776–2.243)	0.306	1.184 (0.688–2.037)	0.542
Other protocol	1.318 (0.402–4.323)	0.648	0.878 (0.259–2.969)	0.834
Type of infertility				
Primary	1		1	
Secondary	1.155 (0.932–1.433)	0.188	1.034 (0.798–1.340)	0.799
Number of embryos transferred				
1	1		1	
2	0.828 (0.663–1.036)	0.099	0.738 (0.503–1.084)	0.121
Stage of embryo transferred				
Cleavage-stage transfer	1		1	
Blastocyst transfer	1.089 (0.857–1.384)	0.485	0.938 (0.762–1.154)	0.542
Previous cesarean section				
No	1		1	
Yes	0.577 (0.357–0.933)	0.025^a^	0.471 (0.275–0.804)	0.006^a^
Endometriosis				
No	1		1	
Yes	0.218 (0.030–1.565)	0.13	0.213 (0.029–1.545)	0.126
PCOS				
No	1		1	
Yes	1.195 (0.886–1.611)	0.243	1.031 (0.749–1.419)	0.853

*ART* assisted reproductive technology, *IVF* in vitro fertilization, *ICSI* intracytoplasmic sperm injection, *EMT* endometrial thickness, *HCG* human chorionic gonadotropin, *GDM* gestational diabetes mellitus, *GnRH* gonadotropin-releasing hormone, *PCOS* polycystic ovary syndrome, *OR* odds ratio, *CI* confidence interval, *aOR* adjusted odds ratio

For variables of age, BMI, estradiol and progesterone level on HCG administration day, restricted cubic splines were generated to adjust for the non-linear relationship between these continuous variables and HDP. Thus, quantitative results were not presented

^a^ *P* < 0.05

## Discussion

In recent years, more and more attention has been paid on the obstetric complications after IVF/ICSI-ET. The aim of this retrospective study was to explore the association between EMT on HCG trigger day and obstetric complications. We examined the predictive value of EMT on clinical outcomes in order to provide a basis for judging the optimal opportunity of ET. A thin endometrial thickness (≤8 mm) was found to be associated with an increased risk of HDP after adjusting for confounding variables, suggesting that the thin endometrium itself is a risk factor for HDP.

HDP is a group of common clinical complications in perinatal period, and a leading cause of maternal morbidity and mortality [30]. Several earlier studies have shown that advanced maternal age, primiparity, twin pregnancy, maternal comorbidities such as chronic hypertension, diabetes, overweight and obesity, and HDP history are risk factors for HDP [31–33]. Therefore, we included only singleton births born to women under the age of 45 years without chronic hypertension or diabetes and adjusted for most of the above risk factors which may affect perinatal outcomes. Moreover, previous cesarean section tended to be a protective factor for HDP, but no previous studies have reported similar findings. The detailed reason for this result is unclear and requires further exploration. In addition to the known causes, the risk factors of HDP and other obstetric complications related to IVF/ICSI-ET parameters need to be further explored. The absence of the corpus luteum [34, 35] and the reduction of immunological tolerance to paternal antigens in semen through surgically obtained sperm [36–38] has been supported to be risk factors for preeclampsia in the literature. And, of course, the EMT has been focused to confirm the predictive value of obstetric complications and perinatal outcomes in recent studies. Our findings are consistent to some extent with one recent study which reported that an EMT < 7.5 mm was associated with an increased risk of obstetric complications in fresh IVF cycles [16]. However, obstetric complications were defined as one or more of the placenta-related pregnancy complications (e.g., preeclampsia, placental abruption, manual removal of adherent placenta, and small for gestational age) in that study, and the separate prevalence of preeclampsia was 5.4 and 4.2% in group EMT < 7.5 mm and group EMT ≥7.5 mm (*P* > 0.05), respectively [16]. By retrospectively analyzing 3157 singleton deliveries, a study of Guo Z et al. [17] showed that the risk of delivering a small for gestational age (SGA) infant was higher in pregnancies with an EMT ≤ 7.5 mm compared with those with an EMT > 12 mm in fresh IVF/ICSI–ET, but no statistically significant differences were found in rates of obstetric complications. A recent research based on 5251 singleton births resulting from FET found that an EMT < 9 mm was associated with an increased risk of placenta previa [19]. He et al. reported that following ART, the risk of PPH was increased significantly when the EMT < 10 mm, and no significant differences were found in HDP, placenta previa and placental abruption [39]. However, there was no statistically significant difference in the rates of both placenta previa and PPH among the three groups in our study about fresh transfer. One possible reason for the discrepancy in different research might exist in the above studied subjects included fresh transfer, frozen transfer, or both, and our study only had fresh transfers.

The clear etiology and pathophysiology of HDP are yet to be elucidated. Preeclampsia is often accompanied by placental hypoperfusion and ischaemia [31, 40, 41]. The main cause of placental hypoperfusion is uterine spiral artery remodeling disorder [42]. Defective deep placentation is one of the possible causes of a spectrum of obstetrical syndromes (including gestational hypertension, preeclampsia, abruption placentae, and spontaneous mid-trimester abortion), which may be associated with restricted remodeling of the spiral arteries in inner myometrium [43]. During normal pregnancy, the remodeling of uterine spiral artery can reach the inner third of the myometrium, and cytotrophoblasts may deeply invade the spiral arteries within the uterine wall. By contrast, in preeclampsia, cytotrophoblast invasion of the uterus is generally shallow. Because spiral artery remodeling fails to reach the myometrium to some degree, the blood flow of placental perfusion was further limited when preeclampsia occurs [44, 45]. Our results suggested that the thin endometrium itself has a significant role in the above-mentioned process and that an appropriate endometrial thickness is important for reducing the incidence of placenta-related pregnancy complications. Further studies are needed to elucidate the histological and molecular mechanism for the effect of thin endometrium on placenta-related pregnancy complications.

To the best of our knowledge, our research is the largest single centre study of its kind, and the first to demonstrate the significant relationship between the thin endometrium and HDP in fresh IVF/ICSI transfer cycles. The EMT of our fertility patients was assessed by trained sonographers of the same team, and the embryos were cultured in the same laboratory conditions, thus the potential bias was minimal. We also recognize that our study has some limitations. Firstly, not all confounders were taken into account due to the retrospective nature of this study. For example, because of the lack of more detailed records, maternal life habit such as smoking were not adequately controlled. A previous cross-sectional study reported that the prevalence of smoking among Chinese pregnant women was only 3.8% [46]. Therefore, the unavailability of smoking data might have minimal impact on the effect estimates in this study. Secondly, maternal age was higher in the thin endometrium group than the other two groups in our study. Several previous studies also reported that older mothers were tended to have a thinner EMT [16, 17, 47]. However, we used an RCS to adjust for age in multivariate analysis, which may achieve a better adjustment of this variable. Thirdly, 68% of the patients transferred two embryos. Therefore, we additionally conducted a subgroup analysis by the number of embryos transferred (Table S1), and results from the two subgroups (one ET and two ET, respectively) were similar with the main results. Thus, the number of embryos transferred may have minimal influence on our results.

## Conclusions

In summary, our data suggested that there is a significant relationship between EMT measured on the day of HCG trigger and HDP in fresh IVF/ICSI–ET cycles, and a thin endometrial lining (EMT ≤ 8 mm) is a risk factor for HDP. However, more prospective cohort studies and mechanism studies are needed to verify our conclusion. Therefore, clinicians should pay close attention to endometrial development for judging the optimal opportunity of ET, and obstetricians should remain aware of the possibility of HDP when women with a thin EMT achieve pregnancy by fresh IVF/ICSI–ET treatment cycles.

## Supplementary Information

**Additional file 1.**

## Data Availability

The datasets used and/or analyzed during the current study are available from the corresponding author on reasonable request.

## Abbreviations

IVF/ICSI: In vitro fertilization/intracytoplasmic sperm injection
ET: Embryo transfer
HDP: Hypertensive disorders of pregnancy
GDM: Gestational diabetes mellitus
PPH: Postpartum hemorrhage
ART: Assisted reproductive technology
EMT: Endometrial thickness
HCG: Human chorionic gonadotropin
PGT: Preimplantation genetic testing
FET: Frozen embryo transfer
GnRH: Gonadotropin-releasing hormone
BMI: Body mass index
PCOS: Polycystic ovary syndrome
ORs: Odds ratios
aOR: Adjusted odds ratio
95% CIs: 95% confidence intervals
ANOVA: Analysis of variance
RCS: Restricted cubic spline.
