# Supplementary material for: Thin endometrium is associated with the risk of hypertensive disorders of pregnancy in fresh IVF/ICSI embryo transfer cycles: a retrospective cohort study of 9,266 singleton births
Source: Reprod Biol Endocrinol. 2021 Apr 9;19:55. doi: 10.1186/s12958-021-00738-9 (PMC8034143; doi:10.1186/s12958-021-00738-9)
Supplement: Supplementary file 1 — Additional file 1. [file 12958_2021_738_MOESM1_ESM.docx]

**Table S1**. Subgroup analysis for univariate and multivariate analysis of EMT on HCG trigger day for HDP by the number of ET.

| **Subgroup** | **Predictor variable** | **OR (95% CI)** | **P value** |  | **aOR (95% CI)** | **P value** |
| --- | --- | --- | --- | --- | --- | --- |
| **One ET** | EMT on HCG trigger day (mm) |  |  |  |  |  |
|  | ≤ 8 | 2.184 (1.062-4.230) | 0.023^a^ |  | 2.077 (1.038-3.988) | 0.018^a^ |
|  | >8-12 | 1 |  |  | 1 |  |
|  | >12 | 1.046 (0.733-1.494) | 0.804 |  | 1.063 (0.742-1.523) | 0.739 |
| **Two ET** | EMT on HCG trigger day (mm) |  |  |  |  |  |
|  | ≤ 8 | 1.687 (1.075-2.646) | 0.023^a^ |  | 1.671 (1.059-2.638) | 0.027^a^ |
|  | >8-12 | 1 |  |  | 1 |  |
|  | >12 | 0.942 (0.660-1.345) | 0.743 |  | 0.971 (0.678-1.392) | 0.971 |

Note: ET: embryos transferred; EMT: endometrial thickness; HCG: human chorionic gonadotropin; OR: odds ratio; CI: confidence interval; aOR: adjusted odds ratio.

Multivariate analysis was adjusted for parity, type of ART treatment, protocol for controlled ovarian stimulation, type of infertility, stage of embryo transferred, previous cesarean section, endometriosis, polycystic ovary syndrome, and restricted cubic splines for age, BMI, estradiol and progesterone level on HCG administration day.

^a^ P <0.05.


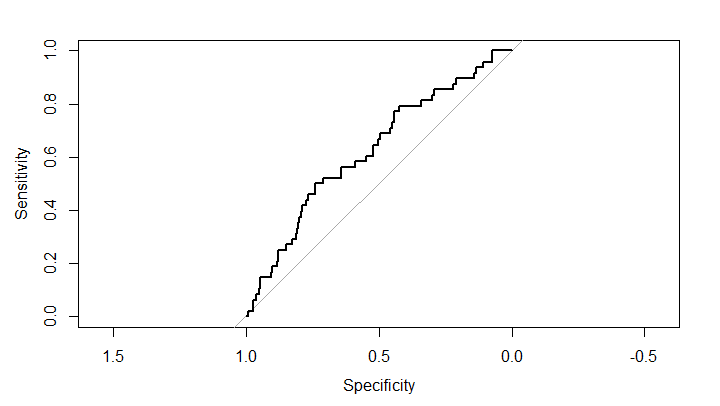


Figure S1. The receiver operating characteristic curve for endometrium discriminating HDP.
